# Mast Cell Activation Triggered by Retrovirus Promotes Acute Viral Infection

**DOI:** 10.3389/fmicb.2022.798660

**Published:** 2022-02-07

**Authors:** Shu-Ting Song, Meng-Li Wu, Hai-Jiao Zhang, Xiao Su, Jian-Hua Wang

**Affiliations:** ^1^Institut Pasteur of Shanghai, Chinese Academy of Sciences, Shanghai, China; ^2^University of Chinese Academy of Sciences, Beijing, China; ^3^Guangzhou Institutes of Biomedicine and Health, Chinese Academy of Sciences, Guangzhou, China; ^4^College of Life Science, Henan Normal University, Xinxiang, China; ^5^State Key Laboratory of Cell Biology, Shanghai Institute of Biochemistry and Cell Biology, CAS Center for Excellence in Molecular Cell Science, Chinese Academy of Sciences, Shanghai, China

**Keywords:** mast cell, degranulation, retrovirus, acute infection, MDSC (myeloid-derived suppressor cell)

## Abstract

Mast cells (MCs) are strategically located at the host-environment interface and their non-allergic roles in the immune-surveillance of pathogens have recently gained more attention. However, MC-caused detrimental regulation of immune inflammations can promote viral invasion. Currently, the role of MCs in retroviral infection remains elusive. We have recently proved that human gut MCs could capture and transfer HIV-1 to CD4^+^ T cells for promoting viral spread; MC-released histamine augments HIV-1-induced functional polarization of dendritic cells to cause immunosuppression via stimulating the differentiation of regulatory T cells. In this study, we used a murine model of MuLV/Friend virus infection to address MC role in acute retroviral infection *in vivo*. The acute infection of MuLV/Friend virus could be established in C57BL/6 wild type mice, but viral acquisition showed low efficiency in C57BL/6-*Kit*^*W*^–^*sh/W*^–^*sh*^ (Sash) mice which lack MCs. In mechanism, we found that MuLV/Friend virus triggered MC activation for degranulation; MC degranulation subsequently activated the granulocyte-like myeloid derived suppressive cells (G-MDSCs) to inhibit CD8^+^ T cells- and NK cells-mediated antiviral immune responses. The reconstruction of MCs in Sash mice promoted acute retroviral infection by regulating G-MDSCs functions and antiviral immune responses. Importantly, the administration of MC stabilizers to block cell degranulation elevated antiviral immune response and consequently suppressed retrovirus infection. This study uncovers a specific role of MCs in acute retroviral infection and elucidates the underlying immune-mechanisms. Targeting MCs may provide a novel approach for controlling acute infection by retroviruses.

## Graphical Abstract

Retrovirus-induced MC degranulation promotes acute viral infection. The acute infection of MuLV/Friend virus triggers MC degranulation, which activates G-MDSCs to inhibit both CD8^+^ T- and NK cell-mediated antiviral immune responses; the HR1 antagonists Eastine and Loratadine that being used as the MC stabilizers block MC degranulation and consequently reverse degranulation-induced immune suppression for viral suppression. G-MDSCs, granulocyte-like myeloid derived suppressive cells; HR1, histamine receptor 1; iNOS, inducible nitric oxide synthase; MC, mast cell.

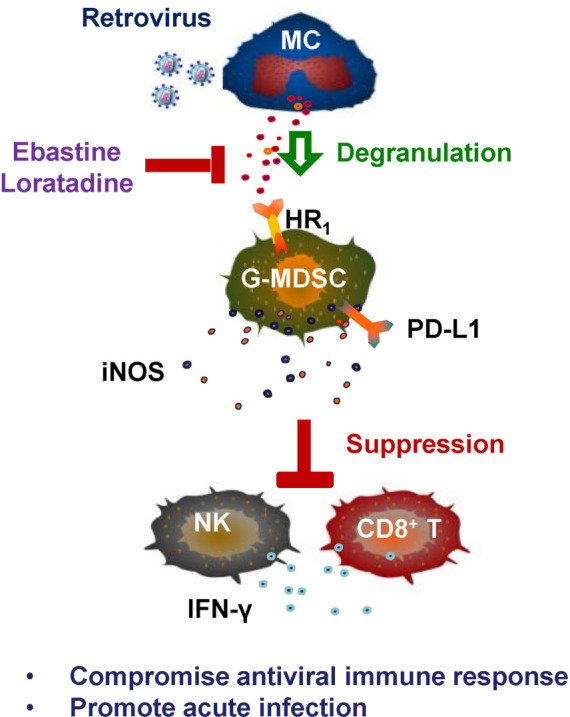

## Introduction

Discovered 140 years ago by Paul Ehrlich, mast cells (MCs) are tissue resident cells strategically located at the host-environment interface, such as skin, airway, gastrointestinal tract, and urinary tract, etc. Historically, MCs are known to cause asthma and allergy through the release of histamine and other factors (Bischoff, 2007). In the last decade, their non-allergic roles in the immune-surveillance of pathogens have gained more attention (Galli et al., 2005; Bischoff, 2007; Abraham and St John, 2010). Upon encountering pathogens, MCs either release a number of granule contents including histamine, heparin, chymotrypsin and antimicrobial peptides to eliminate pathogens, or recruit immune effector cells to kill microbes (St John et al., 2011; Ebert et al., 2014; Pundir et al., 2019).

Through immune surveillance, MCs link innate and adaptive immunity. For instance, the infection of murine cytomegalovirus (CMV) induces MC degranulation and triggers the release of CC chemokine ligand 5 that recruits protective CD8^+^ T cells for viral clearance *in vivo* (Ebert et al., 2014). In dengue virus (DENV) infection, MCs are key sentinels in skin to regulate inflammatory responses locally (Rathore and St John, 2018), and MCs recruit multiple T cell subsets including γδ T cells to the skin and the draining lymph nodes to kill virus-infected cells (Mantri and St John, 2019); MCs stimulate host intracellular antiviral responses through activating the pathways of melanoma differentiation-associated gene 5 (MDA5) and retinoic acid inducible gene I (RIG-I), which induce *de novo* transcription of cytokines that recruit natural killer (NK) and natural killer T (NKT) cells to clear viruses (St John et al., 2011). Upon reovirus invasion, MCs secret cytokines such as IL-10, TNF-a, type I and type III IFNs to enhance NK cell functions (Portales-Cervantes et al., 2017). Based on these immune regulatory functions, MC activator compound 48/80 (C48/80) has been used as the mucosal adjuvant to elicit protective immunity against the infection of influenza virus (Meng et al., 2011; Xu et al., 2014).

Conversely, the detrimental effects of MCs have also been observed in the context of viral infections. The release of pro-inflammatory mediators from MCs causes tissue pathology and promotes viral invasion (St John, 2013; Graham et al., 2015). The released tryptase during DENV virus infection causes the breakdown of endothelial cell tight junctions and disrupts the vascular permeability (Rathore et al., 2019), and the released serotonin promotes platelet activation and aggregation to result in thrombocytopenia (Masri et al., 2019). In Japanese encephalitis virus (JEV) infection, the released chymase disrupts tight-junction proteins and causes breakdown of blood-brain barrier, which result in more viral infection in central nervous system and enhance neurological deficits (Hsieh et al., 2019). MCs released histamine and leukotriene trigger the uncontrolled production of pro-inflammatory factors to induce cytokine storm in mice with influenza A virus (IAV) infection (Graham et al., 2013). The released large amount of pro-inflammatory mediators cause intensive lung injury during H5N1 virus infection (Hu et al., 2012). Of note, some of MC degranulation inhibitors have displayed the demonstrable success in reversing the above pathological situations (Hu et al., 2012; Morrison et al., 2017; Hsieh et al., 2019; Rathore et al., 2019).

The role of MCs in retroviral infection is poorly defined. MCs show accumulation in rectal mucosa and uterine cervix in HIV-1 infected individuals (Bishop et al., 1987; Guimaraes et al., 2011); placental tissue-resident MCs can be infected by HIV-1 and serve as long-lived inducible reservoirs (Sundstrom et al., 2007); we have recently proved that human gut MCs could capture and transfer HIV-1 to CD4^+^ T cells for promoting viral spread (Jiang et al., 2015). These findings indicate that MCs may modulate HIV-1 spread.

To investigate MC function in acute retroviral infection *in vivo*, in this study, we used a murine model of MuLV/Friend virus infection. The mouse model was initially established to understand the basic mechanisms of oncogenesis, then more widely adapted to the study of retrovirus-host interplays as well as innate and adaptive immune responses (Hoatlin and Kabat, 1995; Hasenkrug and Chesebro, 1997; Ney and D’Andrea, 2000; Dittmer et al., 2002; Hasenkrug and Dittmer, 2007; Gibbert et al., 2013; Halemano et al., 2013; Joedicke et al., 2014; Akhmetzyanova et al., 2015; Shen et al., 2018). We found that retrovirus-triggered MC activation for degranulation promotes acute viral infection.

## Materials and Methods

### Assay for MuLV/Friend Virus-Induced Mast Cell Degranulation

For assessing MuLV/Friend virus induced MC degranulation *in vivo*, 6-week-old female C57BL/6 mice were inoculated with MuLV/Friend virus either via i.p., or s.c. injection in footpad with the indicated conditions. Degranulation from splenic or peritoneal MCs was detected with toluidine blue staining or intracellular immunostaining of avidin, and detected by flow cytometry or microcopy. For toluidine blue staining, cells were fixed with 4% paraformaldehyde (Sigma-aldrich) for 10 min at room temperature (RT) and then stained with 1% toluidine blue (Sigma-aldrich) for 1 h at RT. Slides were then washed in distilled water three times and coverslipped with mounting medium. Footpad or spleen tissues were fixed with 4% paraformaldehyde overnight at RT, then dehydrated with 30% sucrose (Sigma-Aldrich), and embedded in Optimum Cutting Temperature Compound (SAKURA). Tissues were cut into 6 μm sections and seeded on slides for immunostaining with specific antibodies and observed under fluorescence microscopy. Histamine in spleen was quantified with an ELISA kits according the manufacturer‘s instructions instruments (Sangon Biotech, D751012).

### Assay for HIV-Induced Mast Cell Degranulation

LAD2 cells were grown in StemPro-34 medium (Gibco) supplemented with 100 μg/mL SCF (Novoprotein), 100 μg/mL IL-6 (Novoprotein), nutrient supplement (NS) (Gibco), 100 U/mL penicillin (Invitrogen), 100 μg/mL of streptomycin (Invitrogen) and 2 mM L-Glutamine (Gibco) at 37°C under 5% CO_2_. LAD2 cell degranulation was evaluated by measuring the release of β-hexosaminidase (Hsieh et al., 2019). Briefly, LAD2 cells (3.5 × 10^5^) were exposed to HIV-JRFL/VLP (10 or 100 ng p24*^Gag^*) or HIV-HXB2/VLP (10 or 100 ng p24*^Gag^*) for the indicated times. C48/80 (4 μg/mL) was used to induce MC degranulation as the positive control. For measuring β-hexosaminidase activity, the substrate of p-nitrophenyl-N-acetyl-β-D-glucosaminide was dissolved in 0.1 M sodium citrate (pH 4.5) for reaction for 1 h at 37°C, then 0.1 M carbonate buffer (pH 10) was added to stop the reaction. The product of 4-p-nitrophenyl was detected at absorbance of 405 nm. β-hexosaminidase was measured in the supernatant as well as the cell lysate solubilized in 0.1% Triton-X100. Percentage of degranulation was calculated by dividing the absorbance in supernatant by the sum of absorbance in both supernatant and cell lysate.

### Assay for Viral Replication

Six-week-old female C57BL/6 or Sash mice were infected i.p. with MuLV/Friend (1 × 10^9^ copies) for indicated times. In some mice, C48/80 (1 mg/kg) was administered along with infection, and Ebastine (5 mg/kg) or Loratadine (10 mg/kg) (both from Sigma-Aldrich) was administered 1 day before infection and treatment was continued each day during infection. At necropsy, spleen, blood or bone marrow (BM) were harvested, and total cellular RNAs were extracted with TRIzol reagent (Life Technologies) and then reverse transcribed to cDNA with ReverTra Ace qPCR RT Master Mix with genome DNA Remover Kit (TOYOBO). Viral replication was quantified by measuring the expression of viral *gag* gene with PCR quantification using the GoldStar TaqMan Mixture (CWBiao). PCR was performed using the Thunderbird SYBR qPCR Mix (TOYOBO) on the ABI 7900HT Real-time PCR system (Applied Biosystems), with an initial denaturation step for 10 min at 95°C, amplification with 40 cycles of denaturation (95°C, 15 s), annealing and extension (60°C, 1 min). The primers for *gag*, forward, 5′-CTC TTT CTC CGA GGA CCC AG- 3′, reverse, 5′-GTC ATT GGG CAG CTG AGT TG- 3′, and the probe: 5′-FAM- ACA GCT TTG ATC GAG TCC GTT CTC CT-TAMRA- 3′.

### Flow Cytometry

The phenotypes of MCs, NK, CD8^+^ T cells, CD4^+^ T cells, G-MDSCs and M-MDSCs were determined by flow cytometry by immunostaining with specific antibodies. The specific monoclonal antibodies against the antigens or isotype-matched IgG controls used were: APC-CD117 (c-Kit) (104D2, Biolegend), PE-FcεR1α (MAR-1; eBioscience), eFluor450-CD3 (145-2C11, eBioscience), APC-NK1.1 (PK136, eBioscience), PE-CD4 (GK1.5, eBioscience), PE-Cyanine7-CD8 (53-6.7, eBioscience), CD16/CD32 (FCR4G8, eBioscience), PE-Cyanine7-CD19 (1D3, eBioscience), PE-Cyanine7-CD3 (145-2C11, eBioscience), PE-Cyanine7-NK1.1 (PK136, eBioscience), PE-Cyanine7-Ter119 (TER-119, eBioscience), PerCP-Cyanine5.5-CD11b (M1/70, eBioscience), APC-Ly6G (1A8, eBioscience), eFluor450-Ly6C (HK1.4, eBioscience), FITC-IFN-γ (XMG1.2, eBioscience), PE-iNOS (CXNFT, eBioscience). For intracellular staining, cells were treated with Intracellular Fixation and Permeabilization Buffer (eBioscience). The stained cells were detected using a Fortessa flow cytometer (BD Pharmingen) and analyzed with FlowJo 7.6.1. software (BD Biosciences).

### The Reconstruction of Mast Cells in Sash Mice

The MC reconstruction in Sash mice was followed the protocol (Wolters et al., 2005), briefly, bone marrow derived mast cells (BMMCs) were generated as follows: bone marrow cells from 8-week-old C57BL/6 mice were harvested and incubated in RPMI-1640 supplemented with 1% non-essential amino acids, 50 mg/L gentamycin, 100 U/mL penicillin, 100 μg/mL streptomycin, 10% fetal bovine serum, 10ng/mL murine IL-3, 30 ng/mL murine stem cell factor and 0.1% 2-mercaptoethanol. Medium was changed every week. Cells were harvested for reconstruction after 3-week incubation. The purity of the BMMCs has a MC phenotype higher than > 90% as determined by flow cytometry immunostaining with FcεRI (BioLegend) and c-Kit (eBioscience). For reconstruction, 3-week-old Sash mouse was injected i.v. with BMMCs (5 × 10^6^) and maintained for an additional 4 weeks before usage.

### Statistical Analysis

Graphpad Prism 7.0 (GraphPad Software) was used for statistical analysis. For direct comparisons of infected WT and Sash mouse samples, Student’s unpaired two-tailed t test was performed to analyze significant differences. For comparisons of multiple groups, Tukey’s multiple comparisons test one-way ANOVAs were performed.

## Results

### Mast Cell Deficiency Shows the Low Efficiency for Retroviral Acquisition

**FIGURE 1:**
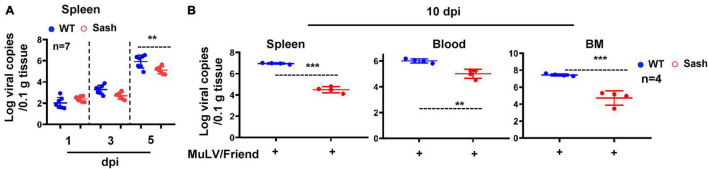
Mast cell deficiency impairs acute retroviral acquisition. C57BL/6 (WT) and Sash mice were infected i.p. with MuLV/Friend (1 × 10^9^ copies) for indicated time and viral replication was quantified in spleen **(A)**, and in multiple tissues at 10 dpi **(B)**. *t*-test was performed to analyze significance differences. Data are presented as means ± SD. **p < 0.01 and ***p < 0.001 are considered significant differences.

To investigate the role of MC in acute retroviral infection *in vivo*, we employed a well-established murine model of Friend murine leukemia virus (MuLV/Friend virus) infection (Hoatlin and Kabat, 1995; Ney and D’Andrea, 2000). We compared the outcomes of intra-peritoneally (i.p.) infection by MuLV/Friend virus between wild type (WT) and MC deficient mice C57BL/6-*Kit*^*W–sh/W*^–^*sh*^ (Sash). Sash mice have the mutations in KIT (a receptor tyrosine kinase protein; also known as Mast/stem cell growth factor receptor), which is the cell surface receptor for the MC growth factor SCF (Galli et al., 2005). Sash mice with MC deficiency showed the low efficiency for viral replication, as demonstrated by the lower viral RNAs in spleen within 5 days of infection (Figure 1A), and the lower viral RNAs in spleen, bone marrow, or peripheral blood at day 10 post infection (Figure 1B). In contrast, higher viral RNAs were detected in all tissues examined in WT mice (Figure 1B). These data demonstrate that the lack of MCs limits retroviral acquisition.

### Mast Cell-Deficiency Increases Both CD8^+^ T- and NK Cells-Mediated Antiviral Immune Responses and Decreases the Accumulation of Active G-MDSCs

**FIGURE 2:**
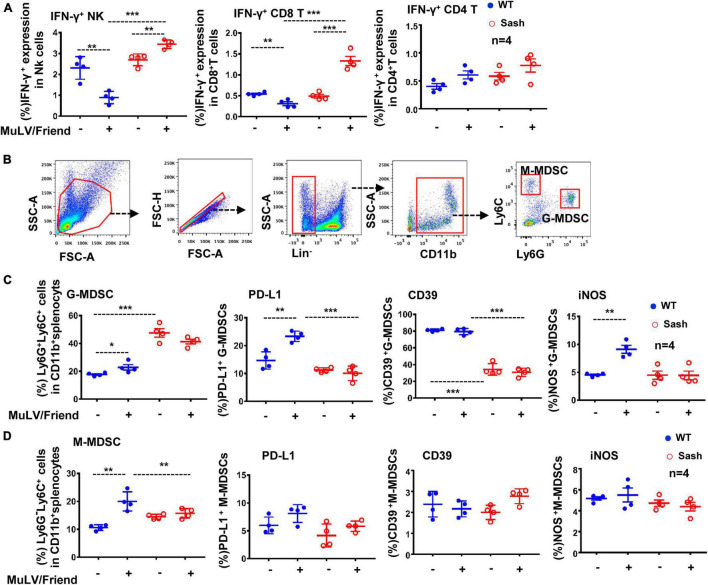
MC-deficiency increases antiviral immune responses and decreases active G-MDSCs accumulation. C57BL/6 (WT) and Sash mice were infected i.p. with MuLV/Friend (1 × 10^9^ copies) for 5 days. **(A)** The IFN-γ expression in these splenic CD4^+^ T, CD8^+^ T and NK cells were measured using intracellular immunostaining and detected by flow cytometry. **(B)** The population of G-MDSCs and M-MDSCs in mice were identified with immunostaining with specific antibodies. **(C,D)** the frequency and the expression of cellular markers of G-MDSCs and M-MDSCs were detected by flow cytometry. Unpaired t-test was performed to analyze significance differences. Data are presented as mean ± SD. *p < 0.05, **p < 0.01 and ***p < 0.001 are considered significant differences.

To explore the possible mechanisms responsible for the limited viral replication in Sash mice, we examined immune cells in spleen that often respond to retroviral infections readily. MuLV/Friend virus infection led to a rapid and progressive depletion of CD4^+^ T cells as expected, and a significant increase of both CD8^+^ T and NK cells in the spleen of both wild type (WT) and Sash mice (Supplementary Figure 1). Notably, viral infection augmented IFN-γ production in both NK and CD8^+^ T cells in Sash mice, but diminished its production in WT mice (Figure 2A); meanwhile, IFN-γ production in CD4^+^ T cells was similar in WT and Sash mice (Figure 2A).

Next, we investigated why the presence of MCs could impair antiviral immune responses. Sash mice are known to have an abnormal large number of MDSCs (Michel et al., 2013), that are a heterogeneous population of myeloid progenitor cells and immature myeloid cells with immune regulatory activities (Gabrilovich and Nagaraj, 2009; Drabczyk-Pluta et al., 2017; Sui et al., 2017), and they have been implicated in multiple pathological conditions including viral infections (Martin et al., 2014; Jachetti et al., 2018). Therefore, we went on to examine whether the distinctive patterns of IFN-γ response in WT mice versus in Sash mice are connected to MDSCs.

The phenotype of MDSCs was readily defined by a combination of markers using flow cytometry analysis (Figure 2B). It is known that mutation in *c-kit* in Sash mice causes aberrant myelopoiesis and accumulation of cells phenotypically and functionally resemble MDSCs in spleen (Michel et al., 2013). Consistent with this, we found that Sash mice have more accumulations of granulocyte-like myeloid derived suppressive cells (G-MDSCs) in spleens in comparison to that in WT mice (Figure 2C). These cells manifest potential suppressive functions by having elevated expression of suppressive markers such as PD-L1, CD39, and iNOS (inducible nitric oxide synthase) (De Santo et al., 2008; Lu et al., 2016; Salem et al., 2017; Zhang et al., 2017; Li et al., 2018). MuLV/Friend virus infection increased the accumulation of both G-MDSCs and monocytic myeloid-derived suppressor cells (M-MDSCs) in both WT and Sash mice, but only elevated the expression of PD-L1 and iNOS on G-MDSCs in WT mice, but not in Sash mice (Figures 2C, D). Of note, although the M-MDSC frequency increased in WT mice upon viral infection, these cells did not have increased expression of suppressive markers (Figure 2D). Taken together, these data demonstrate that MC-deficient mice have the increased both CD8^+^ T- and NK cells-mediated antiviral immune responses and the decreased accumulation of active G-MDSCs; the suppressed anti-viral immune responses in WT mice may account for their susceptibility to retroviruses.

### Mast Cell-Reconstruction in Sash Mice Declines Anti-Viral Immune Responses and Rescues Retroviral Acquisition

**FIGURE 3:**
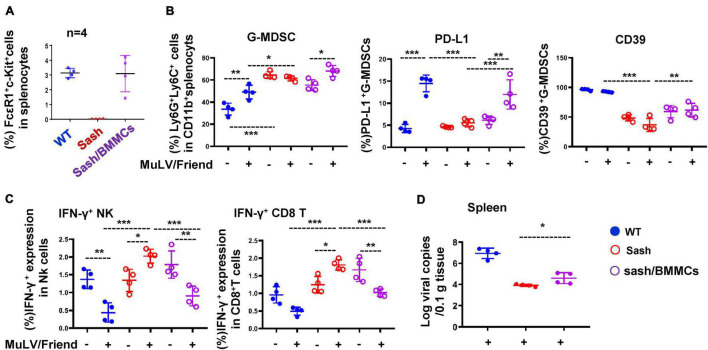
MC-reconstruction in Sash mice declines anti-viral immune responses and rescues retroviral acquisition. **(A)** BMMCs (5 × 10^6^) were injected i.v. into Sash mice to reconstruct MCs. The splenic c-Kit^+^ FcεRI^+^ MCs were quantified. All mice were infected i.p. with MuLV/Friend (1 × 10^9^ copies) for 5 days, **(B)** the frequency and phenotype of splenic G-MDSCs were detected, **(C)** the IFN-γ expression in splenic NK and CD8^+^ T were measured, and **(D)** viral replication in spleen was quantified. One-way ANOVA was performed to analyze significance differences. Data are presented as mean ± SD. *p < 0.05, **p < 0.01 and ***p < 0.001 are considered significant differences.

To confirm the essential role of MCs in promoting acute viral infection, we reconstructed MCs in Sash mice to investigate whether there is a rescue of viral acquisition. The MC reconstruction experiment was performed by i.v. injection of bone marrow-derived mast cells (BMMCs) and then monitored for 4-weeks (Wolters et al., 2005). The MCs were reconstructed in Sash mice (Figure 3A and Supplementary Figure 2). Compared to Sash mice, the MC-reconstruction mice have significantly increased frequency of G-MDSCs and elevated the expression of suppressive markers PD-L1 and CD39 in splenic G-MDSCs after viral infection (Figure 3B, Supplementary Figure 3A), but not in M-MDSCs (Supplementary Figure 3B). Consequently, IFN-γ expression in both NK and CD8^+^ T cells was declined (Figure 3C), and a rescue of viral replication in spleen was observed (Figure 3D). These data confirmed that the presence of MCs is essential for retroviral acquisition.

### Mast Cell Activation for Degranulation Promotes Viral Infection

**FIGURE 4:**
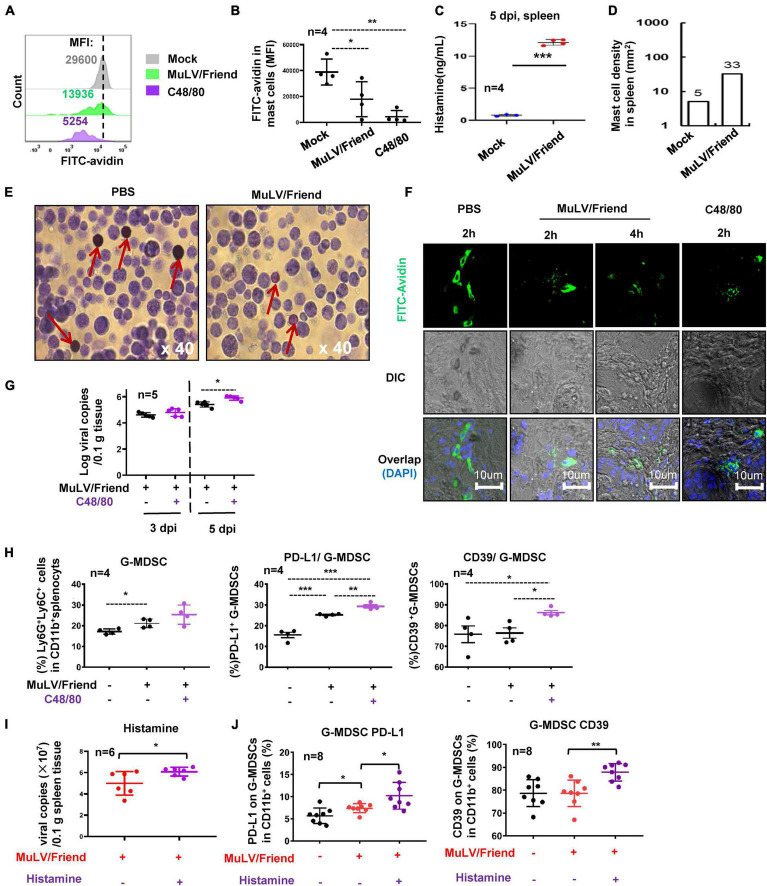
Mast cell activation promotes viral infection. **(A–C)** Virus and C48/80 induces MC degranulation. C57BL/6 mice were treated i.p. with MuLV/Friend (1 × 10^9^ copies) or C48/80 (1 mg/kg) for 2 h. The PBS was used as the mock-infection control. The degranulation from c-Kit^+^ FcεRI^+^ splenic mast cells were measured with the immunostaining of cytoplasmic avidin granules, and the mean fluorescence intensity (MFI) was calculated. The MC degranulation from four mice were summarized **(B)**. The released histamine in spleen at 5 dpi was measured with ELISA **(C)**, and MC accumulation at 5 dpi in the spleen tissue section was calculated **(D)**. **(E)** peritoneal MC degranulation. C57BL/6 mice were infected with MuLV/Friend (1 × 10^9^ copies) for 2 h, and the peritoneal cells were harvested and stained with Toluidine blue to show the degranulation of peritoneal MCs. The red rows indicate MCs. **(F)** Retrovirus-induced MC degranulation in footpad tissue. C57BL/6 mice were s.c. infected with MuLV/Friend virus (1 × 10^8^ copies) in the footpads. The frozen footpad sections were intracellularly immunostained with FITC-avidin to observe MC degranulation. C48/80 (1 mg/kg) was administered along with MuLV/Friend infection of WT mice as above, at 5 dpi, **(G)** viral loads in spleen were quantified, and **(H)** the frequency and phenotype of G-MDSCs in spleen were profiled. WT mice were administrated with histamine (2.5 mg/kg) via i.p. during viral infection as above, at 5 dpi, viral load in spleen was measured **(I)**, and the phenotype of G-MDSCs in spleen were profiled **(J)**. One-way ANOVA was performed to analyze significance differences. Data are presented as mean ± SD. *p < 0.05, **p < 0.01 and ***p < 0.001 are considered significant differences.

We then investigated the mechanism for MC-promoting retroviral infection. We first examined whether acute infection of MuLV/Friend virus could activate MCs. The activation of MCs was determined by measuring cell degranulation, which can be assessed by the intensity reduction of immunostaining of cytoplasmic avidin granules (Zhang et al., 1991; Aalto et al., 1998; Wu et al., 2021). C57BL/6 mice were inoculated i.p. with either MuLV/Friend virus, negative control PBS (mock), or a positive control C48/80 that can activate MCs for inducing degranulation (Chatterjea et al., 2012). After 2 h treatments, both MuLV/Friend virus and C48/80 reduced immunostaining of cytoplasmic avidin granules in c-Kit^+^ FcεRI^+^ splenic mast cells (Figures 4A, B), indicating MC degranulation. The released histamine (Figure 4C) and the accumulation of MCs (Figure 4D) in spleen at 5 dpi were examined. MuLV/Friend virus-induce rapid degranulation was confirmed in the peritoneal MCs. The metachromatic labeling by toluidine blue to indicate MC degranulation was visualized at 2 h post-inoculation (hpi) (Figure 4E). These methods for examination of MC degranulation were applied in the footpad tissues where many MCs reside. Mice were subcutaneously (s.c.) inoculated with the virus or the positive control C48/80 in the footpad, and the rapid MC degranulation was observed upon as early as 2 h treatment with virus or C48/80 (Figure 4F). These data demonstrate that acute retrovirus infection triggers MC activation and degranulation.

To determine whether MC activation can modulate viral acquisition, C57BL/6 WT mice were treated with C48/80 via i.p. during viral infection to induce MC degranulation. C48/80 treatment led to a significantly increased viral replication in spleen at 5dpi (Figure 4G), and concomitant elevated expressions of suppressive markers PD-L1 and CD39 in spleen G-MDSCs (Figure 4H), but not in M-MDSCs (Supplementary Figure 4).

To investigate the contribution of histamine to these phenotypes, the WT mice were administrated with histamine (2.5 mg/kg) via i.p. during viral infection. Histamine elevated viral infection at 5 dpi (Figure 4I), and consistently, the administration of histamine increased the expressions of suppressive markers PD-L1 and CD39 in spleen G-MDSCs (Figure 4J), but not in M-MDSCs (Supplementary Figure 5).

Taken together, these data demonstrate that MuLV/Friend virus -induced MC degranulation promotes virus acquisition.

### Mast Cell Stabilizer Blocks Cell Activation and Reduces Acute Retrovirus Infection

The inflammatory mediators released from MCs can be blocked with a series of MC stabilizers including the higher concentration of second-generation antihistamines (Negro-Alvarez et al., 1996). Ebastine and Loratadine are histamine receptor 1 (HR1) antagonists that are routinely used in the clinics for the treatment of allergy (Goyal et al., 2017; Drummond and Lester, 2018; Keerthana and Vidyavathi, 2018; Sastre, 2020). Ebastine (and its main metabolite carebastine) and Loratadine (and its main metabolite desloratadine) can stabilize MCs to block the release of inflammatory mediators (Yakuo et al., 1994; Wang et al., 2005; Xia et al., 2005; Palikhe et al., 2009; Xie et al., 2017; Gamperl et al., 2021; Wu et al., 2021).

**FIGURE 5:**
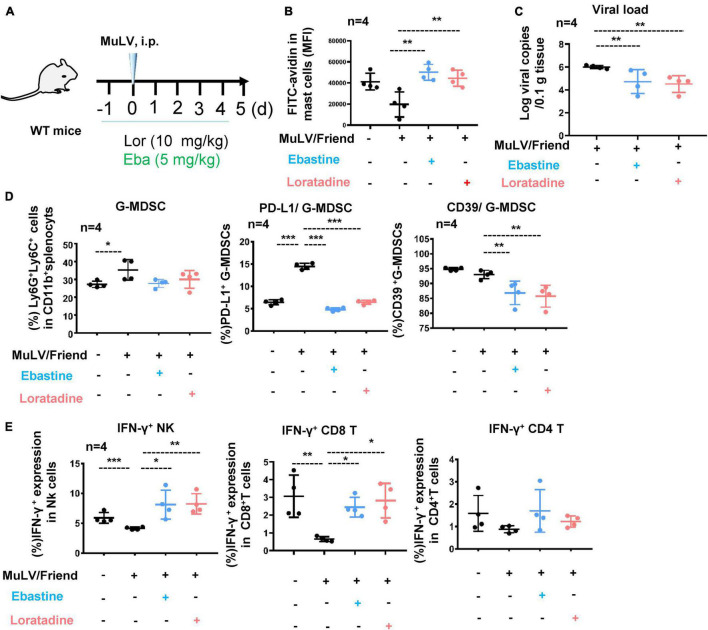
Mast cell stabilizer blocks cell activation and reduces acute retrovirus infection. **(A)** A schematic illustration of infection and treatment. C57BL/6 mice were infected i.p. with MuLV/Friend (1 × 10^9^ copies), and Ebastine (5 mg/kg) or Loratadine (10 mg/kg) was administered 1 day before infection and continued daily throughout infection. At 5 dpi, **(B)** the assay for MC degranulation, **(C)** viral loads in spleen were quantified, **(D)** the frequency and phenotype of splenic G-MDSCs were profiled, and **(E)** the IFN-γ expression in splenic NK, CD8^+^ T and CD4^+^ T cells were measured. One-way ANOVA was performed to analyze significance differences. Data are presented as mean ± SD. *p < 0.05, **p < 0.01 and ***p < 0.001 are considered significant differences.

To confirm that MC activation for degranulation is necessarily required for promoting retroviral acquisition and replication, we blocked MC degranulation by using Ebastine or Loratadine and then detected viral replication. C57BL/6 mice were i.p. administered with Ebastine (5 mg/kg) or Loratadine (10 mg/kg) 1 day before viral inoculation and the treatments were continued daily throughout infection (Figure 5A), and the blocked degranulation from splenic c-Kit^+^ FcεRI^+^ MCs were observed, as indicated by the rescued immunostaining of cytoplasmic avidin granules (Figure 5B). Notably, the administration of Ebastine and Loratadine significantly reduced intracellular viral loads in splenocytes (Figure 5C), indicating that the block of MC degranulation reduced viral replication. Concomitantly, the administration of Ebastine and Loratadine markedly reduced the virus-induced expression of PD-L1 and iNOS in splenic G-MDSCs, but not in M-MDSCs (Figure 5D and Supplementary Figure 6), and greatly improved antiviral IFN-γ responses of both NK cells and CD8^+^ T cells (Figure 5E). Collectively, these findings demonstrate that stabilize MCs to block cell degranulation can elevate antiviral immune response and consequently suppresses retrovirus replication.

### HIV-1 Induces Mast Cell Degranulation

**FIGURE 6:**
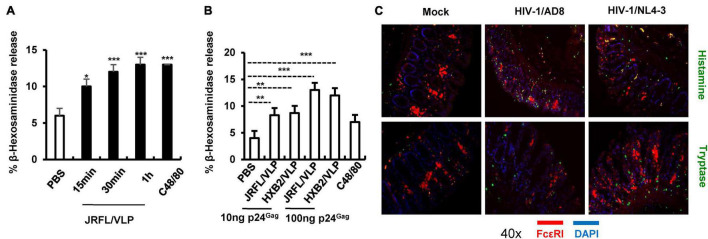
HIV-1-induced MC degranulation. **(A,B)** HIV-1 induced LAD2 cell degranulation. LAD2 cells (3.5 × 10^5^) were exposed to JRFL/VLP (10 ng*^Gag^*) for indicated times **(A)**, or cells were exposed to HIV-JRFL/VLP or HIV-HXB2/VLP for 30 min **(B)**, C48/80 (4 μg/mL) was used as positive controls. LAD2 degranulation was detected by measuring the released β-hexosaminidase. **(C)** *Ex vivo* cultured human colorectal tissues were incubated with HIV-1/AD8 or HIV-1/NL4-3 (4 ng p24*^Gag^* for each) for 16 h, and then snap frozen in optimal cutting temperature. Frozen tissues were sectioned and immunostained with specific antibodies and visualized under confocal microscopy. One-way ANOVA was performed to analyze significance differences. Data are presented as means ± SD. *p < 0.05, **p < 0.01 and ***p < 0.001 were considered significant differences.

Having above demonstrated the MC degranulation triggered by MuLV/Friend virus *in vivo* and its consequence on viral acquisition, the next, we expanded to investigate whether other types of retrovirus particularly HIV-1 could also trigger MC degranulation. LAD2 cells (a human MC cell lines) were treated with HIV-JRFL/VLP [a pseudotyped HIV like particles (VLP) with the HIV-1 CCR5-tropic envelope of JRFL]. Results showed that MC degranulation occurred within 15 min of stimulation, as shown by the secretion of MC granule content β-hexosaminidase (Figure 6A). A parallel experiment using stimulation with HIV-HXB2/VLP (CXCR4 tropic HIV-1 envelope pseudotyped VLP) also induced MC degranulation (Figure 6B). Consistently, a dose-dependent effect of MC degranulation was observed for treatment with both pseudotyped VLPs (Figure 6B).

To more physiologically confirm this, an *ex vivo* tissue culture model was used to investigate whether HIV-1 can induce MC degranulation. Results showed that inoculation of human colorectal tissues with replication competent HIV-1/AD8 (CCR5 tropic) or HIV-1/NL4-3 (CXCR4 tropic) viral isolates for 16 h triggered MC degranulation, as demonstrated by immunostaining patterns and the release of histamine and tryptase (Figure 6C). Taken together, these data demonstrated that HIV-1 can triggers MC degranulation.

## Discussion

The location at mucosa makes MC being the sentinel to the exposed pathogens. By releasing soluble factors or directly cell-to-cell contact, MCs recruit multiple types of immune cells and may beneficially or detrimentally regulate immune inflammation infections (Galli et al., 2005; Orinska et al., 2005; Abraham and St John, 2010; Podlech et al., 2015). In this study, we used the murine model of MuLV/Friend virus infection to uncover the crucial role of MCs in promoting retroviral acquisition, and suggest a MC modulation-based new approach for controlling acute infection by retroviruses.

Our study reveals some cellular and molecular mechanisms through which MCs function during retroviral infection. Upon stimulation by chemical agents or pathogens, MCs can release granule contents that contain stored factors to mediate physiological or pathological functions and *de novo* synthesize more such factors. Our data suggest that the released histamine is a key mediator of the MC function, therefore, the administration of antihistamine drugs Ebastine and Loratadine reverses virus-induce phenotype/functional changes of G-MDSCs and decreases viral acquisition. The detailed mechanisms underlying retrovirus-induced MC degranulation needs further study.

One notable feature of this study is the *in vivo* investigation of the immune regulatory functions of MC in acute retroviral infection. MuLV/Friend virus can infect not only hematopoietic progenitors but also various differentiated immune cells (Hasenkrug and Chesebro, 1997; Dittmer et al., 2002; Akhmetzyanova et al., 2015). Its infection in mice results in chronic viremia, polycythemia, and splenomegaly, erythroleukemia, and death (Chesebro et al., 1990; Li et al., 1990; Hasenkrug and Chesebro, 1997; Miyazawa et al., 2008). The virus induces rapid T-lymphocyte dynamic changes during acute infection, sustains high level of virus replication, and triggers a multitude of antiviral immune responses, thus the mouse model of MuLV/Friend virus infection provides an attractive tool for studying retrovirus infection *in vivo*. Indeed, this mouse model has been effectively used for unraveling HIV-1 pathogenesis and evaluating anti-HIV strategies (Halemano et al., 2013).

Mast Cells are abundantly distributed in the mucosa, the major portal for HIV-1 acquisition and amplification. Regarding to the potential role of MCs in HIV-1 infection, some observational studies have shown that genital mucosa in HIV-infected women contain mast cells with increased density; and that there are more mucosal mast cells in men with AIDS associated diarrhea (Bishop et al., 1987; Guimaraes et al., 2011). These findings may hint that MCs are the risk factors in viral dissemination. For investigating this, we have isolated MCs from human gut tissues and found that these gut MCs could capture and transfer HIV-1 to CD4^+^ T cells for promoting viral dissemination (Jiang et al., 2015). Additionally, we have previously shown that MC-released histamine augmented HIV-1-induced functional polarization of dendritic cell to skew naïve T cell differentiation toward regulatory T cells (Tregs) (Zhai et al., 2013), which contribute to HIV-1-induced immunosuppression. In this study, we found that HIV-1 could trigger MC degranulation in cell- and tissue-culture based models. It would be interesting to investigate HIV-1-induced MC degranulation *in vivo* and the consequent effects on viral mucosal acquisition. The model of HIV-1 infection in humanized mice or mucosal SIV infection in macaques provide the suitable tools.

In summary, using the murine model of MuLV/Friend virus infection, we demonstrate the essential role of MCs in retroviral acquisition; virus-induced MC degranulation promotes viral infection, and the HR1 antagonists that being used as MC stabilizers block cell degranulation and consequently suppress the establishment of retrovirus infection. Our findings provide a potential new approach for prevention of acute infection by retroviruses.

## Data Availability Statement

The original contributions presented in the study are included in the article/Supplementary Material, further inquiries can be directed to the corresponding author/s.

## Ethics Statement

The studies involving human participants were reviewed and approved, and the human colorectal tissues have been received from an already-existing collection (Jiang et al., 2015). In that previous collection, written informed consent was provided by study participants, and the study was approved by the Institutional Ethical Committee of The First Affiliated Hospital of Nanjing Medical University, Nanjing, China. The patients/participants provided their written informed consent to participate in this study. The animal study was reviewed and approved, and all mice operation procedures were conducted in compliance with a protocol approved by the Institutional Animal Care and Use Committee at Institute Pasteur of Shanghai, Chinese Academy of Sciences (SYXK-Shanghai- 2018-0039).

## Author Contributions

J-HW: conceptualization. S-TS, M-LW, and H-JZ: data curation, formal analysis, and visualization. J-HW and S-TS: writing-review and editing. J-HW and XS: supervision, and project administration and funding acquisition. All authors contributed to the article and approved the submitted version.

## Conflict of Interest

The authors declare that the research was conducted in the absence of any commercial or financial relationships that could be construed as a potential conflict of interest.

## Publisher’s Note

All claims expressed in this article are solely those of the authors and do not necessarily represent those of their affiliated organizations, or those of the publisher, the editors and the reviewers. Any product that may be evaluated in this article, or claim that may be made by its manufacturer, is not guaranteed or endorsed by the publisher.
