# Supplementary material for: Mast Cell Activation Triggered by Retrovirus Promotes Acute Viral Infection
Source: Front Microbiol. 2022 Feb 7;13:798660. doi: 10.3389/fmicb.2022.798660 (PMC8859150; doi:10.3389/fmicb.2022.798660)
Supplement: Supplementary file 1 [file Data_Sheet_1.pdf]

Mast cell activation triggered by retrovirus promotes acute viral infection

Supplementary Figure legends

Figure S1. Immune cells population. C57BL/6 and Sash mice were infected i.p. with MuLV/Friend (1×10^9 copies) for indicated times, the frequencies of $CD4^+$ T, $CD8^+$ T and NK cells in spleen were quantified. One-way ANOVA was performed to analyze significance differences. Data are presented as mean ± SD. * $p<0.05$, ** $p<0.01$ and *** $p<0.001$ are considered significant differences.

Figure S2. BMMCs reconstruction. BMMCs (5×10^6) were injected i.v. into Sash mice to reconstruct MCs. The splenic c-Kit^+ $Fc\varepsilon RI^+$ MCs were quantified. Data from one representative mouse are shown.

Figure S3. MDSCs profile. Mice of WT, Sash and Sash/BMMCs were infected i.p. with MuLV/Friend (1×10^9 copies) for 5 d, (A) the expressions of PD-L1 and CD39 in G-MDSCs were measured with flow cytometry. Data from one representative mouse are shown, (B) the frequency and phenotype of splenic M-MDSCs were profiled. One-way ANOVA was performed to analyze significance differences. Data are presented as mean ± SD. * $p<0.05$ and *** $p<0.001$ are considered significant differences.

Figure S4. M-MDSCs in C48/80-treated infected mice. C57BL/6 mice was administered with C48/80 (1 mg/kg) along with MuLV/Friend i.p. infection (1×10^9 copies). At 5dpi, the phenotypes of splenic M-MDSCs were profiled. One-way

ANOVA was performed to analyze significance differences. Data are presented as mean ± SD. *** $p<0.001$ is considered significant differences.

Figure S5. Phenotypes of M-MDSCs in histamine-treated infected mice. WT C57BL/6 mice were administrated with histamine (2.5 mg/kg) via i.p. during viral infection as above, at 5 dpi, the phenotypes of M-MDSCs in spleen were profiled.

Figure S6. Phenotypes of M-MDSCs in Ebastine- or Loratadine-treated infected mice. C57BL/6 mice were infected i.p. with MuLV/Friend (1×10^9 copies), and Ebastine (5mg/kg) or Loratadine(10mg/kg) was administered 1 day before infection and continued daily throughout infection. At 5 dpi, the phenotypes of M-MDSCs in spleen were profiled. One-way ANOVA was performed to analyze significance differences. Data are presented as mean ± SD. ** $p<0.01$ is considered significant differences.

Figure S1

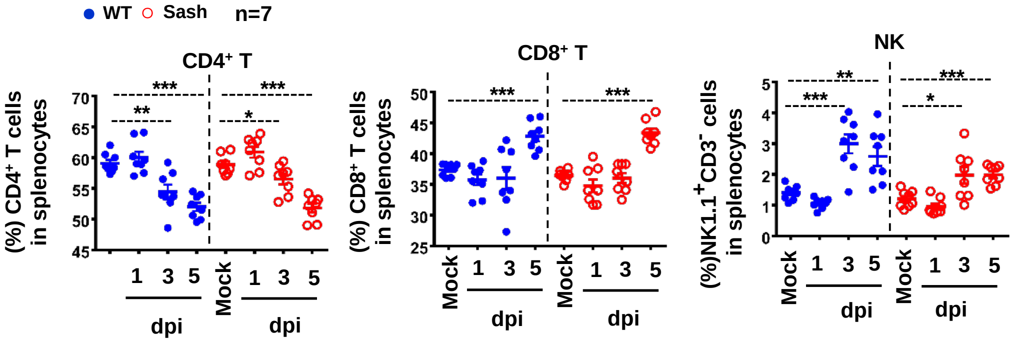

WT
Sash
n=7
CD4+ T
(%) CD4+ T cells in splenocytes
70
65
60
55
50
45
***
**
***
*
1
3
5
Mock
1
3
5
dpi
dpi
CD8+ T
(%) CD8+ T cells in splenocytes
50
45
40
35
30
25
***
***
Mock
1
3
5
Mock
1
3
5
dpi
dpi
NK
(%)NK1.1+CD3- cells in splenocytes
5
4
3
2
1
0
**
***
***
*
Mock
1
3
5
Mock
1
3
5
dpi
dpi

Figure S2

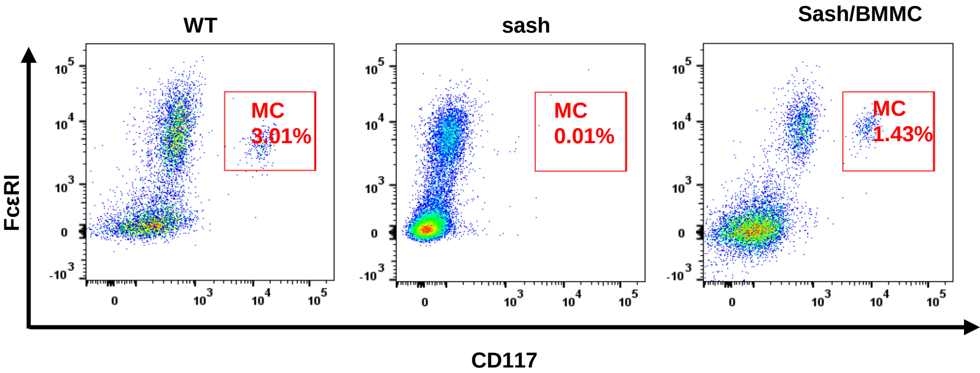

WT
sash
Sash/BMMC
MC
3.01%
MC
0.01%
MC
1.43%
FcεRI
CD117
10^5
10^4
10^3
0
-10^3

Figure S3

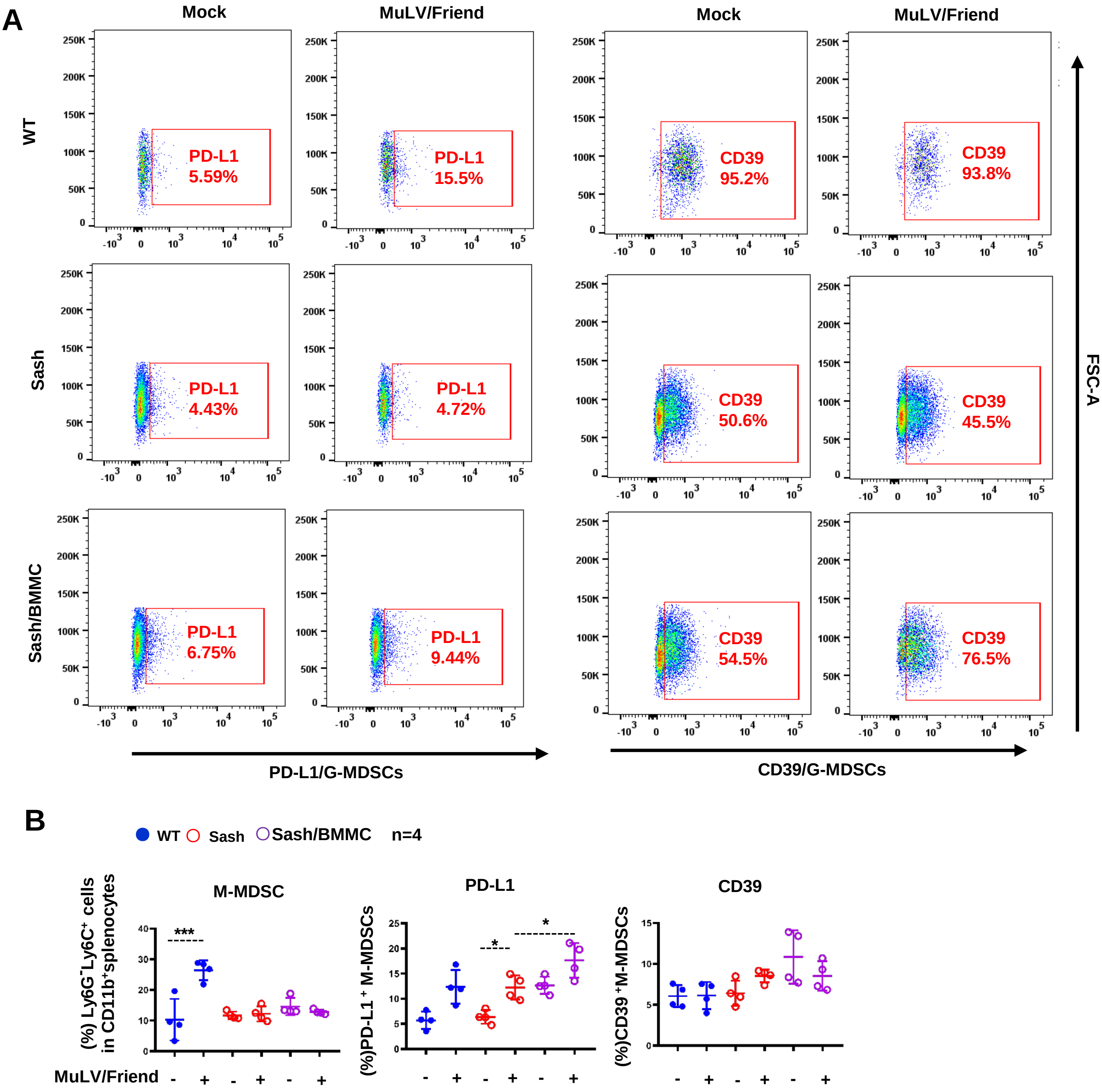
A
Mock
MuLV/Friend
Mock
MuLV/Friend
WT
Sash
Sash/BMMC
PD-L1 5.59%
PD-L1 15.5%
CD39 95.2%
CD39 93.8%
PD-L1 4.43%
PD-L1 4.72%
CD39 50.6%
CD39 45.5%
PD-L1 6.75%
PD-L1 9.44%
CD39 54.5%
CD39 76.5%
FSC-A
PD-L1/G-MDSCs
CD39/G-MDSCs
B
WT Sash Sash/BMMC n=4
M-MDSC
PD-L1
CD39
(%) Ly6G-Ly6C+ cells in CD11b+splenocytes
(%)PD-L1+ M-MDSCs
(%)CD39+M-MDSCs
MuLV/Friend - + - + - +
***
*
*

Figure S4

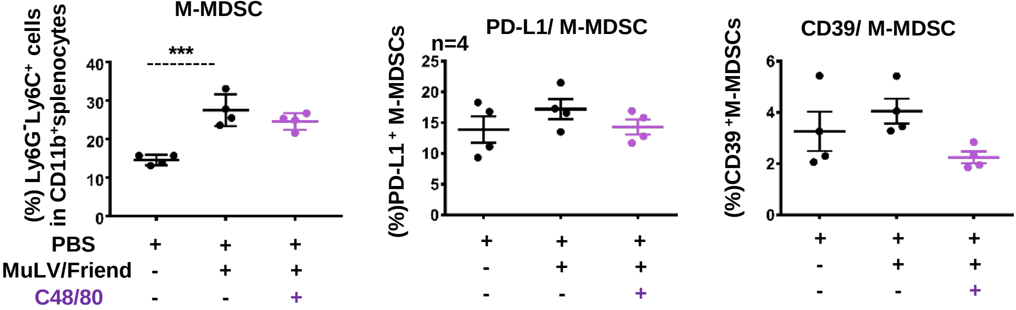

M-MDSC
(%) Ly6G⁻Ly6C⁺ cells in CD11b⁺splenocytes
***
0
10
20
30
40
PD-L1/ M-MDSC
n=4
(%)PD-L1 ⁺ M-MDSCs
0
5
10
15
20
25
CD39/ M-MDSC
(%)CD39 ⁺M-MDSCs
0
2
4
6
PBS + + +
MuLV/Friend - + +
C48/80 - - +

Figure S5

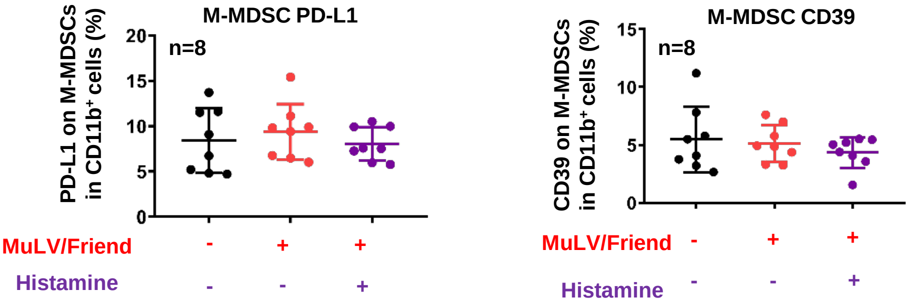

M-MDSC PD-L1
PD-L1 on M-MDSCs in CD11b⁺ cells (%)
n=8
20
15
10
5
0
M-MDSC CD39
CD39 on M-MDSCs in CD11b⁺ cells (%)
n=8
15
10
5
0
MuLV/Friend - + +
Histamine - - +
MuLV/Friend - + +
Histamine - - +

Figure S6

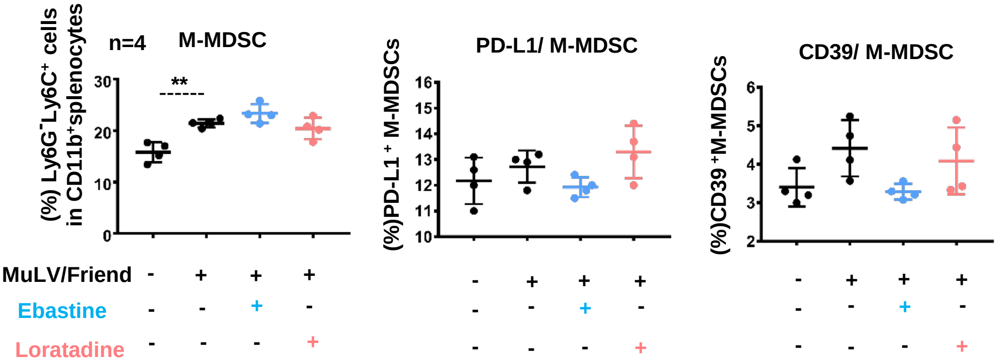
n=4
M-MDSC
PD-L1/ M-MDSC
CD39/ M-MDSC
(%) Ly6G⁻Ly6C⁺ cells in CD11b⁺splenocytes
(%)PD-L1⁺ M-MDSCs
(%)CD39⁺M-MDSCs
**
MuLV/Friend - + + +
Ebastine - - + -
Loratadine - - - +
